# Exoskeleton Training for Spinal Cord Injury Neuropathic Pain (ExSCIP): Protocol for a Phase 2 Feasibility Randomised Trial

**DOI:** 10.12688/hrbopenres.13949.1

**Published:** 2024-09-02

**Authors:** Conor White, Orlaith Doherty, Eimear Smith, Catherine Blake, Nanna Brix Finnerup, Nathan Kirwan, Mark Pollock, Olive Lennon

**Affiliations:** 1 UCD School of Public Health, Physiotherapy and Sports Science, Health Sciences Centre, University College Dublin, Dublin, Leinster, Ireland; 2 National Rehabilitation Hospital, Dún Laoghaire, County Dublin, Ireland; 3 Royal Hospital Donnybrook, Dublin, Leinster, Ireland; 4 Mater Misericordiae University Hospital, Dublin, Leinster, Ireland; 5 Aarhus University Department of Clinical Medicine, Aarhus, Central Denmark Region, Denmark; 6 Helpful Steps Charity, Cork, Ireland; 7 Mark Pollock Trust, Dublin, Ireland

**Keywords:** Spinal Cord Injuries, Neuropathic Pain, Robotics, Adult, Neurorehabilitation

## Abstract

**Background:**

Following Spinal Cord Injury (SCI), 53% of people develop neuropathic pain (NP). NP can be more debilitating than other consequences of SCI, and a persistent health issue. Pharmacotherapies are commonly recommended for NP management in SCI, although severe pain often remains refractory to these treatments in many sufferers. Furthermore, poor medication adherence exists, stemming from unacceptable side-effects and fear of dependency.

Sensorimotor stimulation using active walking with robotic assistance has not been well studied in NP after SCI, despite convincing locomotor-based pre-clinical studies, identifying prevention and reversal of NP.

Our primary aim is to assess the impact of exoskeleton-based walking on NP intensity and interference after SCI and examine feasibility outcomes for progression to a definitive trial.

**Methods:**

This is a phase 2 single-blinded, randomised feasibility study. It will test the feasibility and acceptability of exoskeleton-based walking 3 times per week for 12 weeks (intervention), as a mechanistic-based intervention for NP after SCI. The comparator will be an equally dosed, blended relaxation programme devoid of motor imagery prompts. 40 participants with moderate-to-severe NP post SCI will be recruited and randomised to intervention and comparator groups.

The primary outcomes are feasibility outcomes for progression to definitive trial which include recruitment and retention rates, adverse events and acceptability of the intervention.

Secondary outcomes explore changes in NP intensity and interference as measured by the International Spinal Cord Injury Pain Basic Data Set 3.0 (ISCIPBDS) at baseline, post-intervention (week 13) and at 6-month follow-up.

**Conclusions:**

There is a need to explore non-pharmacological management of NP after SCI. The findings of this feasibility trial will inform the development of a future multicentre, international RCT,

**Trial Registration:**

NCT06463418, 08/07/2024, https://clinicaltrials.gov/study/NCT06463418.

## Introduction

### Background and rationale

Traumatic spinal cord injury (SCI) incidence in Ireland is 11.5-13.3 per million per year ( Smith *et al.*, 2018), placing it as a rare condition. However, the SCI itself in many cases results in significant physical disability and poses a considerable functional and economic burden at an individual and societal level ( McDaid *et al.*, 2019). Pain (nociceptive and neuropathic) is frequently experienced after SCI. Neuropathic pain (NP) type, commonly subdivided into at-level and below level pain, is the most distressing. Following SCI, approximately 53% of people develop neuropathic pain (NP) ( Burke *et al.*, 2017; Finnerup *et al.*, 2014). National SCI data identify high pain intensity and pain interference levels with NP presentations and significantly poorer quality of life (QoL) when compared with other pain phenotypes ( Burke *et al.*, 2018; Burke *et al.*, 2019). Individuals have described NP as more debilitating than the other consequences of SCI ( Hearn *et al.*, 2015), as their most persistent health issue and adequate pain relief as an unmet need ( Kennedy *et al.*, 2006). The mainstay of NP treatment after SCI is pharmacotherapy ( Loh *et al.*, 2022). Pregabalin/gabapentin, duloxetine, amitriptyline and/or opioids are the first- and second-line treatments recommended ( Loh *et al.*, 2022), with high use of non-steroidal anti-inflammatories and paracetamol also reported in survey data ( Burke *et al.*, 2019; Cardenas & Jensen, 2006; Jensen *et al.*, 2005). Severe pain remains refractory to these treatments in 2⁄3 sufferers ( Guy *et al.*, 2016) and significant, often intolerable, central nervous system side-effects are reported ( Siddall *et al.*, 2006). These, together with fear of medication dependency, result in poor adherence to pharmacological regimens leading to a call for non-pharmacological treatment options for people with NP after SCI ( Cardenas & Jensen, 2006; Löfgren & Norrbrink, 2012; Siddall *et al.*, 2006; Widerström-Noga *et al.*, 2020).

The intensity of the NP may increase over time after SCI to become a chronic and life-long condition ( Finnerup *et al.*, 2014; Siddall, 2009). NP following SCI that emerges within the first year after SCI and tends to become chronic is characterised by sensory deficits, spontaneous or stimulus-evoked pain, including allodynia and hyperalgesia and may be associated with dysesthesia and paraesthesia. Qualitative studies, exploring people’s experiences with NP in daily life after SCI ( Buscemi *et al.*, 2018; Widerström-Noga *et al.*, 2017) emphasise the difficult nature of pain, its treatment resistance, and its deleterious effects on QoL. They further identify the need to gain more knowledge and skills in relation to self-management and coping with pain to reduce its impact on their daily life ( Henwood *et al.*, 2012; Norrbrink & Löfgren, 2016). The need for better information regarding pain and additional treatment options has been highlighted in the literature by people with SCI, calling for a stronger focus on persistent pain as a consequence of SCI, better access to healthcare providers with expertise in this area ( Norman *et al.*, 2010). International data identify the proportional burden of NP following SCI as significant. Ninety-four percent of individuals with SCI NP are prescribed >1 medication, the mean number of physician office visits in a 6-month period due to SCI NP is reported as 2 and the total annualised cost of NP per subject in the United States (US) is reported as $26,270 (direct $8,636, indirect $17,634) ( Mann *et al.*, 2013). The presence of pain is further associated with lower return to work rates following injury, and more than a third of individuals with SCI in employment report frequent pain interference with their work ( Widerström-Noga *et al.*, 2001). Pain interference with function, health status and work are noted to be significantly worse in individuals with more severe NP (P<0.0005), where overall work impairment is reported at 38% ( Widerström-Noga *et al.*, 2001).

NP after SCI is multi-faceted and heterogenous, making isolation of specific mechanisms more challenging. Nonetheless, proposed mechanisms include neuronal hyperexcitability (central and peripheral sensitisation) and corticothalamic maladaptive neuroplasticity ( Finnerup *et al.*, 2009). Additionally, NP symptom severity post SCI has been reported to be associated with a combination of residual spinothalamic tract (STT) function below the level of injury and with catastrophising pain coping mechanisms ( Widerström-Noga *et al.*, 2016). Our understanding of the mechanistic effects of sensorimotor stimulation on NP in SCI stem from Phantom Limb Pain research where significant reversal of cortical dysfunction in the primary somatosensory cortex is evident ( Foell *et al.*, 2014). Similar maladaptive cortical reorganisation is hypothesised to be associated with NP in SCI ( Flor, 2008; Wrigley *et al.*, 2009), supported by data garnered from electroencephalography (EEG) studies identifying thalamo-cortical dysrhythmia ( Boord *et al.*, 2008; Jensen *et al.*, 2013; Sarnthein & Jeanmonod, 2008; Vuckovic *et al.*, 2018a) and decreased reactivity in alpha band power signals to eye opening ( Boord *et al.*, 2008; Vuckovic *et al.*, 2014). Thus, EEG may have application as a biomarker both for current NP and as a predictor of NP development in SCI ( Mussigmann *et al.*, 2022; Vuckovic *et al.*, 2018b).

Virtual reality (immersive virtual walking/virtual illusion/imagined walking) has shown promise for reducing NP intensity and interference after SCI ( Austin & Siddall, 2021; Gustin *et al.*, 2023; Trost *et al.*, 2022). Virtual illusion interventions show evidence of direct and corrective stimulation to maladaptive sensorimotor reorganisation in SCI patients with NP ( Eick & Richardson, 2015; Moseley, 2007), supporting the theory that NP mechanisms are reversible. However, actual sensorimotor intervention studies are inconclusive in SCI at this point ( Nees *et al.*, 2017) with limited focus on walking despite compelling preclinical studies showing prevention and /or reversal of SCI neuropathic pain ( Dugan & Sagen, 2015; Dugan *et al.*, 2021; Hutchinson *et al.*, 2004). Notably in these animal studies, other exercise modalities such as swimming and stance training had short term or no effect on NP after SCI. This is suggestive of the fact that the characteristics of walking not seen in swimming and stance training, namely the rhythmic stimulation of proprioceptive and mechanosensory afferents combined with weight bearing might be the potential mechanisms that are driving this reduction in NP ( Dugan *et al.*, 2021).

Robotic-assisted gait training after SCI is well established in the neurorehabilitation space, notably for motor impairment and gait outcomes in incomplete SCI ( Alashram *et al.*, 2021; Stampacchia *et al.*, 2022). However, its mechanistic effects on NP for individuals with SCI have not been well investigated to date. Only two case studies/series employing robotic-assisted gait were identified in the extant literature that mandated participants have NP in the moderate and severe categories prior to using the exoskeleton and results demonstrated meaningful reductions in pain intensity ratings ( Cruciger *et al.*, 2016; Kressler *et al.*, 2014). The ExSCIP randomised feasibility trial addresses the current lack of clinical trials in this area, examining exoskeleton-based walking 3 times per week, as a mechanistic-based intervention for NP after SCI. It will test the feasibility and acceptability of an exoskeleton-based walking intervention, and whether it demonstrates positive signals in reduction of NP intensity and interference levels to warrant onward progression to a definitive trial.

### Aims and objectives

The overall aim of this study is to examine the feasibility and acceptability of an exoskeleton, mechanistic-targeted, walking intervention for NP in people with SCI.

The primary objectives for the study are:

1. Implement an exoskeleton training programme for people with below level NP > 6 months after a traumatic SCI.
2. Pilot and assess the impact of an exoskeleton-based walking intervention in NP > 6 months after SCI, examining feasibility outcomes and short and long-term (6-months) changes in pain intensity and pain interference.

## Methods

### Study design and setting

The ExSCIP study is a single centre, phase 2 randomised, single blinded, feasibility trial, informed by Bowen’s framework for feasibility studies ( Bowen *et al.*, 2009). Given SCI is considered a rare condition, we will apply a randomised feasibility study to allow a better-informed decision on whether to proceed to a larger multi-centre and international trial ( Eldridge *et al.*, 2016). This trial protocol has been registered on clinicaltrials.gov (NCT06463418) https://clinicaltrials.gov/study/NCT06463418)

Progression criteria are based on consideration of the primary objectives around feasibility and the potential for effectiveness and implementation in clinical practice. Quantitative and qualitative process evaluation data will be analysed to consider the following continuation criteria.

- Successful uptake, recruitment, and retention.
- Successful implementation of the ExSCIP intervention.
- Process evaluation indicates that ExSCIP is acceptable to people with NP after SCI and to staff delivering the intervention.
- A positive effect on pain and pain interference outcomes are identified and are meaningful.

The intervention will be delivered in the Motion Analysis Laboratory at University College Dublin (UCD).

### Participants

Participants will be adults who have sustained a traumatic SCI with confirmed below-level NP. Participants will be recruited from the Spinal Injuries Ireland (SII) national support service via information leaflet and trial advertisement and the outpatient SCI clinic at the National Rehabilitation Hospital (NRH) via advertisement fliers and posters. Interested parties will contact the researcher directly to proceed to the next stage of assessment/screening for the trial as outlined below. Eligibility criteria for participants can be found in Table 1.

**Table 1.** Participant Eligibility Criteria.

Inclusion Criteria	Exclusion Criteria
Individuals aged 18 years and older	Individuals aged < 18 years old
Confirmed traumatic SCI of > 6 months duration with complete or incomplete paraplegia or tetraplegia. This must be confirmed by registration at the National Spinal Cord System of Care Programme.	Non-traumatic SCI, cauda equina lesions (excluding fracture at L1/2 causing paraplegia) or Guillain Barré diagnoses.
Below-level NP (≥ 3 levels below neurological level and/or extending to at-level region) starting after the SCI, persisting for >3 continuous months, despite pharmacotherapy ( Bryce *et al.*, 2012) matching the ISCIP Pain Classification and confirmed by neurological examination, and a score of ≥4 on the Douleur Neuropathique 4 (DN4) Bouhassira *et al.*, 2005).	Nociceptive pain profiles only based on subjective pain history and clinical examination.
Moderate to severe NP levels, confirmed as pain ≥ 3 (moderate) and ≥ 6 (severe) on the 0-10 Numerical Rating Scale (NRS) for NP (averaged over a week) included in the ISCIPBDS 3.0 ( Widerström-Noga *et al.*, 2023).	NP intensities of <3 (0-10 NRS)
Exoskeleton naïve.	Anthropometric measurements incompatible with the exoskeleton device (i.e. height >1.9m, weight >100kgs, significant lower limb spasticity).
Stable pain and spasticity medication regimen (i.e. no medication changes for ≥3 months).	Unstable comorbid medical condition/ psychiatric condition/medication regimen.
Have the capacity to provide informed consent.	Planned surgery coinciding with the intervention.
	Pregnancy.
	Substance use disorders.

### Below-level neuropathic pain screening protocol

***Stage 1: Telephone screening***

NP will be screened for as a minimum criterion initially by phone. This phone screening will do the following:

- Confirm their SCI diagnosis (e.g. traumatic aetiology and >6 months post injury).
- Confirm they are on a stable medication regimen.
- Confirm they are exoskeleton naïve.
- Screen for the presence and location of NP supported by the use of the Spinal Cord Injury Pain Instrument (SCIPI) ( Bryce *et al.*, 2014). Score of ≥ 2 is indicative of probable NP in SCI. (Permission has been obtained for reuse of this instrument).
- Screen for anthropometric exclusion criteria.
- Once the phone screening has been completed, candidates deemed to be potentially suitable to participate pending an in-person assessment. Potential participants will be provided with a participant information leaflet at this point and given a minimum one-week grace period to provide informed consent agreeing to undergoing an in-person assessment and to participate in the study.

***Stage 2: In-Person assessment***

Once informed consent is obtained from candidates, an in-person assessment will be performed by an independent assessor. The assessment will entail the following steps:

**Confirmation of presence of moderate to severe below level NP:**

- NP will be confirmed based on a neurological examination, a score of ≥4 on the Douleur Neuropathique 4 (DN4) ( Bouhassira *et al.*, 2005) and a comprehensive pain history. (Permission has been obtained for reuse of this instrument).
- This will be supported using the ISCIP Pain Classification ( Bryce *et al.*, 2012).
- Moderate and severe NP as confirmed above will be described as pain ≥ 3 and ≥ 6 on the 0-10 Numerical Rating Scale (NRS) for NP (averaged over a week). (Permission has been obtained for reuse of this instrument).

**Anthropometric and clinical assessment for compatibility for use of exoskeleton:**

- Participants will undergo an anthropometric and clinical assessment to ensure no height, weight, joint range of movement or muscle spasticity restrictions to exoskeleton use apply.

***Stage 3: Data collection***

**Figure 1.**
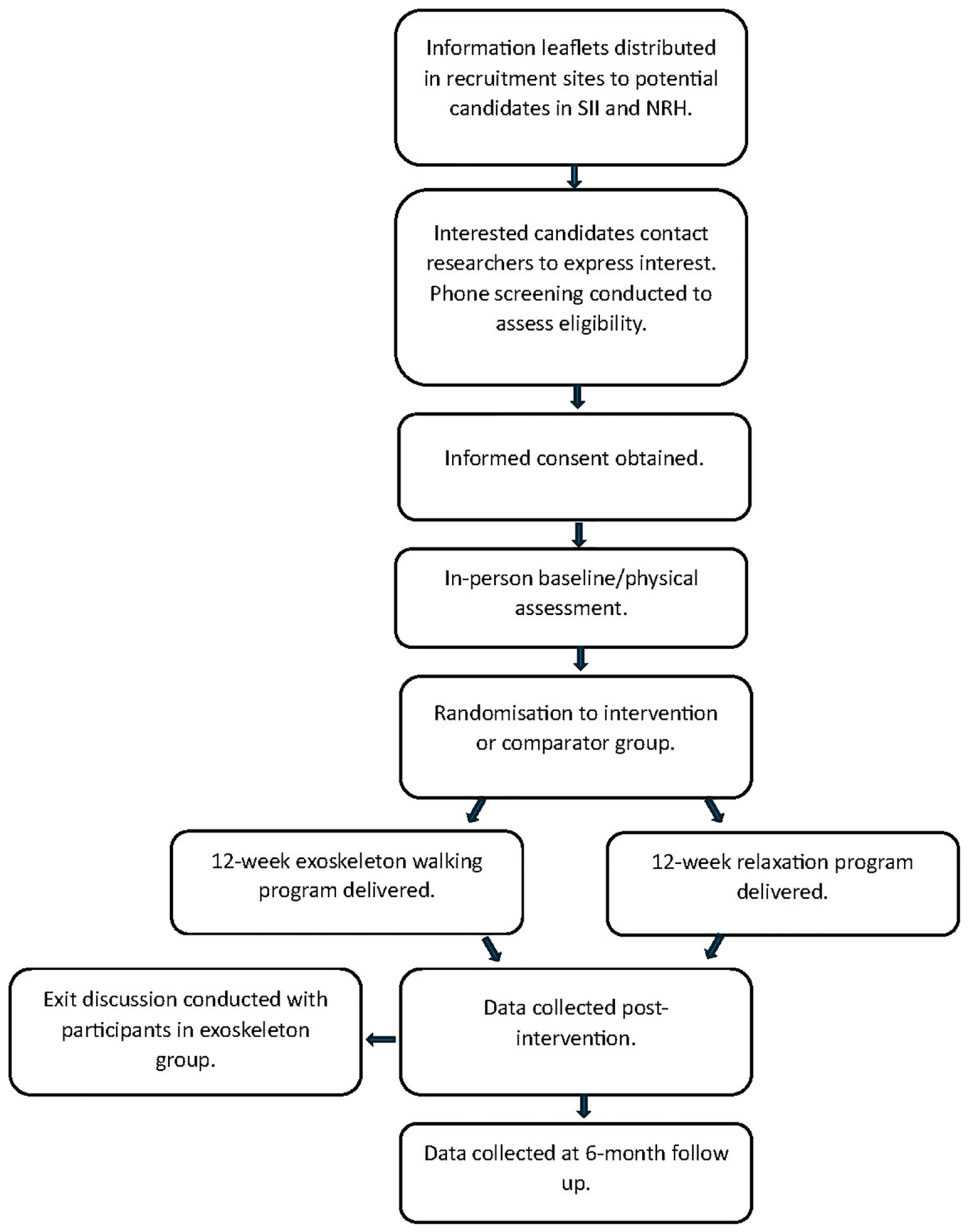
Participant flow through study.

- The independent assessor will collect baseline data for candidates who meet inclusion criteria for the following:
  ◦ Social and health demographics (gender, age, years since injury, neurological level of injury, ASIA impairment scale)
  ◦ Full ISCIPBDS 3.0 for NP intensity and interference measures ( Widerström-Noga *et al.*, 2023). (Permission has been obtained for reuse of this instrument).
  ◦ Neuropathic Pain Symptom Inventory (NPSI) ( Bouhassira *et al.*, 2004; Wong *et al.*, 2020). (Permission has been obtained for reuse of this instrument).
  ◦ EQ-5D-5L ( Herdman *et al.*, 2011) (Permission has been obtained for reuse of this instrument).
  ◦ Resting EEG recordings will be recorded with the participants eyes open for 3 minutes and eyes closed for 3 minutes (NeuroCONCISE device), with participants seated in a dimly lit room shielded from sound and stray electric. Figure 1 details the flow of participants during the course of the study.

- For full details of data collection and management procedures a data management plan for the study has been published ( https://dmp.ucd.ie/public_plans)

### Sample size, randomisation, and blinding

As ExSCIP is a feasibility trial, a sample size calculation will not be conducted. The trial aims to recruit 20 participants to the intervention arm and 20 to the control arm, retaining at least 32 at follow-up. Due to practical considerations including traumatic SCI being a rare condition in Ireland, the strict eligibility criteria in addition to the precedent set by previous RCT’s in this area with considerably smaller sample sizes ( Martinez *et al.*, 2018), this number is considered adequate to inform whether sufficient participants can be recruited and retained in the study and to evaluate the feasibility goals outlined.

Following informed consent and baseline evaluation for exoskeleton, recruited participants will then be randomly allocated to the intervention group (ExSCIP) or to the comparator group (receiving a blended relaxation programme devoid of motor imagery). This will be completed by an independent gatekeeper based in the UCD Centre for Support and Training in Analysis and Research (CSTAR), using a pre-generated electronic randomisation schedule stratified by NP severity. Allocation disclosure will be made by telephone by the independent gatekeeper in CSTAR to the trial interventionist after informed consent and baseline evaluation.

To minimise potential biases, all assessments and outcome measures (baseline, post intervention and 6-month follow-up) will be taken by an independent assessor blinded to the treatment allocation. The data analyst will also be blinded to the intervention allocation.

Blinding participants to the ExSCIP intervention is not feasible. The hypothesis of the study will be masked however in the information to subjects. Participants will be informed that the trial is comparing two types of treatment, to avoid higher expectations in the ExSCIP group.

### Description of intervention and comparator

Participants will be randomised to either:

**Intervention (ExSCIP) group:** the intervention group will complete an exoskeleton-based, mechanistic-targeted, progressive walking programme consisting of one-hour sessions of robotic walking 3 times a week for 12 weeks with hands-on supervision provided by a trained, CORU-registered physiotherapist in the UCD Motion Analysis Laboratory. Those randomised to the intervention group will undergo an initial familiarisation trial walking session in the device under the direct supervision of the PI, to optimise device settings to each individual.

Rest periods will be allowed as required and, as individual aerobic capacity will differ, the programme will be individually tailored to increase the number of steps taken and the number of consecutive steps taken without rest during progressive sessions. Total steps taken and the number of overall scheduled sessions attended will be recorded.

**Control group:** the control group will complete an equally dosed blended relaxation programme, devoid of any motor imagery prompts. It will comprise one face-to-face relaxation class per week alongside 2 online classes over a 12-week period.

Participants in both groups will be permitted to continue with their usual care regimen throughout the duration of the trial. They will not be permitted to participate in additional exoskeleton interventions outside of those provided as part of this study.

### Outcomes

***Primary outcomes***

The primary outcomes will focus on trial feasibility. Full details of these and progression criteria to a multicentre, international RCT can be found in Table 2:

**Table 2.** Feasibility Outcomes and Progression Criteria.

Feasibility Outcomes	Progression Criteria
Number of individuals who contact researchers about the trial.	Successful intervention uptake, with recruitment rates of ≥35% of individuals deemed eligible following in-person assessment and at least 20 individuals randomised to the two arms.
Recruitment rates disaggregated by SII and NRH channels (recruitment capacity at named sites).	Loss to follow-up/attrition rates < 20%.
Number of individuals screened by phone.	Qualitative data that indicates that ExSCIP is an acceptable intervention to individuals with NP following SCI and to physiotherapists delivering the intervention, where no insurmountable barriers were identified.
Number of individuals who are eligible to proceed to in person-assessment following phone screening.	Safe implementation of the ExSCIP intervention: no serious adverse events
Number of individuals who consent to participate in the study following phone screening.	Average programme adherence of ≥ 80% of all sessions.
Trial uptake rates.	A non-inferior effect for NP intensity is observed in the intervention arm in comparison to the control arm.
Number who are eligible to participate in the study after in-person assessment.	A Minimal Clinically Important Difference (MCID) for NP intensity in an SCI population (1.74 points decrease on NRS) is observed within the 95% confidence interval for mean change for the intervention arm ( Farrar *et al*., 2001).
Number randomised to participate in the study.	
Adherence with the 12-week exoskeleton walking programme (attendance and total number of steps taken)	
Any safety/adverse events.	
Feasibility of stratified randomisation	
Number who are lost to follow up at week 13 and 6-month follow up.	
Feasibility of assessment procedures.	
Time needed to collect and analyse data.	

***Secondary outcomes***

**Pain**

Changes in NP intensity will be measured using the ISCIPBDS 3.0 pain intensity scale ( Widerstrom-Noga *et al.*, 2023). Changes in NP interference as measured in the ISCIPBDS 3.0 pain interference scale and changes in neuropathic pain symptoms will be assessed using the Neuropathic Pain Symptom Inventory ( Bouhassira *et al.*, 2004; Wong *et al.*, 2020). In addition, we will use resting EEG as a biomarker for chronic NP. All pain related outcomes will be measured at three timepoints: baseline (week 1/Timepoint 1 (T1)), post intervention (week 13/Timepoint 2 (T2)) and at 6-month follow-up (Timepoint 3 (T3)).

**Health Related Quality of Life (HRQoL)**

We will assess HRQoL using the EUROQOL EQ-5D-5L ( Herdman *et al.*, 2011) at baseline, post-intervention and 6-month follow-up.

### Qualitative study

Qualitative focus group discussions with participants and interventionists will be conducted following the intervention to ascertain their experiences of the ExSCIP programme. As pain is multidimensional and often influenced by factors such as attitudes, self-efficacy and thoughts, inclusion of a qualitative study is important to inform additional development and improvement to the intervention.

The qualitative semi-structured question schedule will be developed in collaboration with our PPI panel. A person unknown to the participants will complete the exit discussions. Key areas to be discussed will be:

- satisfaction with and acceptability of the ExSCIP programme,
- perceptions of exoskeleton use (positive and negative)
- barriers and facilitators to attendance, implementation and continuation of the intervention.
- perceptions of pain assessment tools and whether they feel it captures their pain.

Qualitative feedback will also be obtained from staff who delivered the ExSCIP intervention to evaluate physiotherapists’ perspectives of delivering an exoskeleton walking programme for pain, with questions addressing.

- Beliefs
- Views on implementation
- Therapist burden
- Skill level required to deliver an intervention of this nature.

Qualitative analysis will be underpinned by the principles of phenomenology: open-ended responses from focus group discussions and debriefing interviews will be subject to reflexive thematic analysis. Audio recorded data will be transcribed verbatim by an independent person and transcripts read by two researchers independently. Participant research codes will be used in transcription.

### Data/statistical analysis

Baseline demographics of group participants will be presented in two manners. Categorical data will be presented as frequencies & percentages. Continuous data will be presented as means & standard deviations. If there is evidence of skew in continuous data, mean and standard deviation (SD) will be substituted with median and interquartile range.

Outcome variables will be assessed at three time points (baseline, week 13, 6 month follow up). Health related quality of life (HRQoL) as assessed by the EQ-5D-5L will be converted from value scores to index scores for each item which will allow for calculation of a collated index score ( Hobbins *et al.*, 2018). Pain intensity, pain interference and HRQoL index scores will be presented as mean and SD at each time point. Within group mean differences and 95% confidence intervals (95% CI) will be calculated at week 13- and 6-month follow-up compared to baseline. Between group differences at each time point will be calculated using repeated measures ANOVAs. The effect sizes of between group differences will be assessed using ETA squared/Omega squared statistics. Statistical significance will be determined α-priori at an alpha level of 0.05. Statistical analysis will be undertaken using IBM SPSS version 29 ( IBM, 2023) and conducted as intention-to-treat and per-protocol. Alternatively, this statistical analysis can be performed using the open access R software ( R core team, 2024).

Exploratory EEG data analysis will use a multilevel linear mixed model (LMM) to examine differences between the intervention groups over time in the EEG alpha, beta and theta band power. Repeated measures within participants will be modelled as a random effect. Fixed effects in the model, will include group assignment and time. The moderating effects of pain intensity and interference will also be evaluated. The LMM will study both main effects and interaction effects using the R package lme4 ( Bates *et al.*, 2015) to fit the models. Models will be compared using Likelihood Ratio Tests (LRT) to assess the significance of effects.

### Trial governance and data monitoring

A Trial Steering Committee (TSC) and Trial Management Group (TMG) are established to ensure good governance, trial management and safety monitoring for the duration of the study. The UCD Clinical Research Centre (CRC) team will support the management and conduct of the trial including the development and maintenance of trial documentation according to Good Clinical Practice (GCP) guidelines and UCD CRC Standard Operating Procedures (SOPs).

An independent data monitoring committee (DMC) will also be formed comprising an independent statistician and two independent nominees by the CRC at UCD. They will monitor emergent data in relation to safety and efficacy. Its terms of reference will outline clearly stopping rules and the frequency of the interim data analysis during the recruitment phase. Any adverse events will be monitored by this committee where they will review each event and decide if it is related, might be related or is unrelated to the intervention.

### Safety and adverse events

Risk identification and minimisation is essential in an exoskeleton-based walking intervention delivered to a population that is primarily sedentary. Clear exclusion criteria for exoskeleton use will be applied to those who are not suitable for the device use. A trial will be provided prior to programme commencement to further determine suitability and acceptability and appropriate staff training and safety harnessing will be applied.

Adverse events and near misses will be logged throughout the study period and reported to the Data Management Committee, including the date it occurred, the circumstances, assistance provided or onward medical referral (i.e. medical attention required) and what was implemented to prevent this occurring again.

The Project Manager and PI will be advised of each event immediately and will determine what strategies need to occur based on the severity of the event. Serious adverse events (SAEs) will be reported to the SAE oversight committee and the trial will be paused pending investigation.

SAEs will be reported to the UCD HREC within 15 days of occurrence. In relation to COVID-19 and other community transmissible diseases, we will follow public health advice and HSE policies in relation to infection control measures when the study starts.

### Dissemination of findings

Study findings will be published in appropriate peer-reviewed journals. We will also present study results at relevant national and international conferences (e.g. Irish Pain Society annual conference, Irish Association of Rehabilitation Medicine Annual Scientific Conference and International Spinal Cord Society Conference)

Our PPI Panel will support with knowledge exchange and dissemination activities that include the preparation of plain language summaries of the study results and their implications for people with spinal cord injury and their families, discussion groups, short video clips, social media messages, media interviews and leveraging existing websites of the PPI panel at Spinal Injuries Ireland (spinalinjuries.ie), Mark Pollock Foundation (markpollock.com) and Helpful Steps Charity (helpfulsteps.ie). We will work with these charities to coordinate knowledge exchange and the dissemination of the study findings, alongside key stakeholders in the organisation and delivery of SCI services in Ireland (the National Spinal Cord System of Care (SCSC) Programme at the NRH and the National Spinal Injuries Unit, Mater Hospital).

### Patient Public Involvement

Our PPI Panel was instrumental in developing the grant application for this study and the research protocol, informing both the intervention & control. The panel will continue to work with the researchers during the conduct of the study, working collaboratively to develop the recruitment materials, the interview schedule for the qualitative study and will assist in future dissemination, determining the media, plain language used, and approaches taken in dissemination of findings, including their implications for people with SCI and their families, discussion groups. As co-researchers they will have representation on the Trial Steering committee.

### Study status

This study is currently ongoing. The expected study end date is November 2026.

## Discussion

This study protocol describes the methods of a phase-2, randomised feasibility trial assessing the effects of an exoskeleton- walking as a mechanistic-based intervention for neuropathic pain intensity and interference after spinal cord injury. There have been consistent calls for non-pharmacological treatments for NP after SCI ( Löfgren & Norrbrink, 2012; Widerstrom-Noga *et al.*, 2020). This study aims to contribute to the evidence-base of non-pharmacological management of NP after SCI with a view to informing clinical practice. By examining the feasibility and acceptability of a 12-week exoskeleton walking program for NP after SCI, it will inform us whether progression to a definitive, international, multicentre trial is warranted. This is augmented by the use of a qualitative analysis of participants’ experiences of this trial as well as barriers and facilitators to participation. This will enable us to refine the ExSCIP intervention accordingly for a multicentre trial should feasibility outcomes be met.

## Ethical and consent

Ethical approval has been provided by the UCD Human Research Ethics Committee (HREC) (Approval Number: LS-22-24-White Lennon; date of approval: 7 ^th^ June 2024) and the NRH HREC (Date of approval: 18 ^th^ July 2024). Informed consent will be obtained following provision of the participant information leaflet and explanation of the study and form legal basis for personal data use. Participant consent forms will detail opt-in consent for each proposed use of participant data. Where possible, informed consent will be obtained through written means. Where it is not possible to obtain informed consent through written means, it will be obtained verbally with a family member present as a witness. Both means of obtaining informed consent have been approved by both above mentioned HRECs.

## Data Availability

At the end of the trial, the master sheet linking participants personal details with their trial code will be destroyed and anonymised files will be deposited in a secure data repository (Zenodo) with a permanent identifier (DOI), available to the wider research community under a CC0 license. Participants will have an opportunity to consent or not to anonymised data archiving. **TiDieR Checklist** (DOI: 10.5281/zenodo.13135173) *https://zenodo.org/records/13135173* **SPIRIT Checklist** (DOI: 10.5281/zenodo.13135455) *https://zenodo.org/records/13135455* **Participant Information Leaflet** (DOI: 10.5281/zenodo.13124714) *https://zenodo.org/records/13124715* **Model Consent Form** (DOI: 10.5281/zenodo.13124412) *https://zenodo.org/records/13124413* **Data Management Plan** ( https://dmp.ucd.ie/public_plans) This protocol is written in line with SPIRIT and TIDieR guidelines for reporting outcomes in trial protocols ( Chan *et al.*, 2013; Hopewell *et al.*, 2022; Hoffmann *et al.*, 2014). See extended data for completed checklists. Version 1 (29/07/2024).
