# Supplementary material for: Exoskeleton Training for Spinal Cord Injury Neuropathic Pain (ExSCIP): Protocol for a Phase 2 Feasibility Randomised Trial
Source: HRB Open Res. 2024 Sep 2;7:55. [Version 1] doi: 10.12688/hrbopenres.13949.1 (PMC11748425; doi:10.12688/hrbopenres.13949.1)
Supplement: Peer review history

# Reviewer report: Reviewer response for version 1

**DOI:** 10.21956/hrbopenres.15305.r44121

**Published:** 2025-01-17

**Authors:** Jeff Petruska

**Affiliations:** 1 University of Louisville, Louisville, KY, USA

The topic of the study is of great importance. Neuropathic pain is a major issue for many populations, but those living with spinal cord injury have a particular burden of pain, and much of it is resistant to standard pharmacological treatment. Activity-based therapies have shown great promise for many aspects of the post-SCI condition.

 The protocol is well written, thoughtful, and encompassing. It proposes a feasibility study intended to aid in design of larger studies, or determine that such studies are not feasible or necessary. This is a strength because the proposed intervention is not easily administered or widely available, making this type of feasibility study necessary to establish the parameters for a more thorough study and their priorities.

 Some considerations:

 It is not clear if there is a plan to categorize participants for signal continuity from below-level to brain. This may matter because inclusion criteria include both complete and incomplete injuries.

 There is strength in the well-established methods and outcomes for interventions for locomotion. These can serve as an internal control outcome measure – patients locomotor outcomes can be expected to adhere to similar range, etc., demonstrating the efficacy of the activity.

 Unclear what constitutes being “exoskeleton naïve”. Never used? Are unaware of their existence? Etc. The purpose of this criterion is not clear.

 Participants blinded to hypothesis is a strength

 This participant/caregiver group tends to be socially close-knit. Unclear if efforts will be made to minimize participant cross-talk about the program, or if this is not necessary. Blinding to hypothesis may alleviate the need.

 Outcomes and Progression is strong

Is the study design appropriate for the research question?

Yes

Is the rationale for, and objectives of, the study clearly described?

Yes

Are sufficient details of the methods provided to allow replication by others?

Yes

Are the datasets clearly presented in a useable and accessible format?

Not applicable

Reviewer Expertise:

Neuroscience, neurophysiology, neuroanatomy, sinal cord injury, nerve injury, pain

I confirm that I have read this submission and believe that I have an appropriate level of expertise to confirm that it is of an acceptable scientific standard.

# Reviewer report: Reviewer response for version 1

**DOI:** 10.21956/hrbopenres.15305.r44126

**Published:** 2025-01-07

**Authors:** Hui Zhong

**Affiliations:** 1 Rancho Research Institute, Downey, CA, USA

The protocol, "Exoskeleton Training for Spinal Cord Injury Neuropathic Pain (ExSCIP)," presents a detailed framework for a randomized feasibility trial investigating exoskeleton-based walking as a non-pharmacological intervention for neuropathic pain (NP) in spinal cord injury (SCI) patients.  It is thorough and well written.  Below are some recommendations for improving the protocol:

 1, For ExSCIP group, it was stated in the protocol that total number of steps taken and the number of overall scheduled sessions attended will we recorded.  Please clarify the analysis approach for step counts and session attendance in relation to study outcomes.

 2, Expand details on how adverse events (AEs) and serious adverse events (SAEs) will be analyzed and integrated into feasibility criteria.

 3, When initiating weightbearing activities like exoskeleton stepping in a patient with chronic SCI particularly those with complete injuries, the risk of fracture should be addressed. 

 4, Elaborate on the rationale for using EEG alpha, beta, and theta power as biomarkers for NP. Provide a supplementary explanation linking these metrics to hypothesized changes in neuropathic pain mechanisms.  Clarify how EEG findings will inform conclusions regarding the intervention’s efficacy.

 5, Consider including a preliminary framework for assessing the intervention’s cost-effectiveness relative to standard care and relative to inclusion of surgically invasive spinal implanted devices. This will enhance the clinical applicability and potential impact of the findings.

Is the study design appropriate for the research question?

Yes

Is the rationale for, and objectives of, the study clearly described?

Yes

Are sufficient details of the methods provided to allow replication by others?

Yes

Are the datasets clearly presented in a useable and accessible format?

Not applicable

Reviewer Expertise:

Neuromodulation, Spinal cord injury

I confirm that I have read this submission and believe that I have an appropriate level of expertise to confirm that it is of an acceptable scientific standard, however I have significant reservations, as outlined above.

# Reviewer report: Reviewer response for version 1

**DOI:** 10.21956/hrbopenres.15305.r42477

**Published:** 2024-10-22

**Authors:** Adalberto Loyola-Sanchez

**Affiliations:** 1 Division of Physical Medicine and Rehabilitation, Department of Medicine, Faculty of Medicine & Dentistry, University of Alberta, Edmonton, Alberta, Canada

This is a clearly written protocol of a mixed methods study that includes a Phase 2 RCT and a qualitative reflexive thematic analysis methodologies to determine the feasibility of conducting a trial to assess the effectiveness of exoskeleton walking training on reducing neuropathic pain for individuals with spinal cord injuries. 

 I completed a revision of this protocol based on the Standard Protocol Items: Recommendations for Intervention Trials (SPIRIT) check list and I think the protocol adequately describes most of the fundamental parts of an intervention trial. 

 I do have the following minor edit suggestions that can improve the quality of this manuscript: 

 1) Study design and setting. I recommend to clarify in this section that this is a mixed methods explanatory design (see Creswell et al definition) formed by a quantitative phase followed by a qualitative phase. The quantitative phase consists of the single blinded RCT while the qualitative phase will be a reflexive thematic analysis (provide reference for which approach of reflexive thematic analysis authors will implement, is this the Plan and Clark proposal?) based on a phenomenology informed world view aimed at understanding experiences of participating in the quantitative phase of the study.

 2) Table 1- I recommend to remove the first 4 exclusion criteria as they are the opposite of the inclusion criteria. Typically exclusion criteria are applied to the population that fulfils inclusion criteria. Therefore, only the following are really exclusion criteria: Anthropometric measurements incompatible with the exoskeleton device (i.e. height >1.9m, weight >100kgs, significant lower limb spasticity), Unstable comorbid medical condition/psychiatric condition/medication regimen, Planned surgery coinciding with the intervention, Pregnancy and Substance use disorders.

 3) Exclusion criteria. I also recommend to explain why psychiatric conditions, including substance use disorders are considered as exclusion criteria. This potentially represents an inequity situation for this population so the ethical justification for excluding them is essential to be transparently disclosed.

 4)Secondary Outcomes. Pain. As a clinician I am quite intrigued by the selection of EEG as a biomarker for NP. I recommend to add a brief explanation on how this biomarker works as a NP outcome, I assume is linked with the animal-model research described in the Justification, but I think it is important to clarify this further.

 5) Safety and adverse events- I recommend to briefly mention how participants who may suffer harm from using the exoskeleton will be compensated or attended.

Is the study design appropriate for the research question?

Yes

Is the rationale for, and objectives of, the study clearly described?

Yes

Are sufficient details of the methods provided to allow replication by others?

Yes

Are the datasets clearly presented in a useable and accessible format?

Not applicable

Reviewer Expertise:

Mixed methods, community based rehabilitation, benchmarking trials, spinal cord injury

I confirm that I have read this submission and believe that I have an appropriate level of expertise to confirm that it is of an acceptable scientific standard, however I have significant reservations, as outlined above.
